# Predictors of Pathologic Response After Total Neoadjuvant Therapy in Patients With Rectal Adenocarcinoma: A National Cancer Database Analysis

**DOI:** 10.7759/cureus.17233

**Published:** 2021-08-16

**Authors:** David M McDermott, Sarah A Singh, Paul B Renz, Shaakir Hasan, Josh Weir

**Affiliations:** 1 Radiation Oncology, West Virginia University School of Medicine, Morgantown, USA; 2 Radiation Oncology, Allegheny Health Network, Pittsburgh, USA; 3 Radiation Oncology, New York Proton Center, New York, USA

**Keywords:** total neoadjuvant therapy, colo rectal cancer, national cancer database and seer analyses, non metastatic colo-rectal, chemoradiation therapy

## Abstract

Purpose/objectives

Induction chemotherapy followed by chemoradiation and surgical resection in rectal cancer, known as total neoadjuvant therapy (TNT), is associated with improved pathologic complete response (pCR) rates. The National Cancer Database was utilized to identify factors associated with pCR and survival following treatment with TNT compared to standard neoadjuvant chemoradiation (nCRT).

Materials/methods

The National Cancer Database was queried from 2004 to 2015 for patients with locally advanced, non-metastatic rectal cancer. We identified 16,299 patients receiving neoadjuvant chemotherapy and radiation followed by definitive surgical resection. Patients were stratified by treatment received, either TNT (n=350) or nCRT (n=15,949). Multivariate binomial regression analysis and propensity matching were used to evaluate predictors of pCR. Kaplan-Meier and Cox multivariate analysis of survival were performed.

Results

Median follow-up was 38 months vs 53 months in the TNT vs nCRT groups, respectively. There were more patients with T4 or node-positive disease in the TNT group. There was a trend towards improved pCR in the TNT group (p=0.053). Patients achieving pCR had improved 5-year overall survival (OS) of 85.1%. The 5-year OS was not improved for TNT (76.2%) over nCRT (69.9%) (p=0.19). Pelvic nodal pCR was significantly higher in the TNT group (72%). When stratified by clinical stage, patients with cT3 (p=0.038) or cN1 (p=0.049) disease had improved OS with TNT.

Conclusions

Compared to nCRT, TNT is correlated with higher rates of complete pelvic nodal clearance in patients with locally advanced rectal adenocarcinoma. The use of TNT showed improved survival in patients with cT3 and cN1 disease, indicating a potential benefit for patients with less advanced disease.

## Introduction

Nearly 50,000 people are diagnosed with rectal cancer every year in the United States and colorectal cancer is the third leading cause of cancer death nationwide [1]. The treatment paradigm for stage II-III locally advanced rectal cancer involves neoadjuvant chemoradiation (nCRT) followed by surgery and adjuvant chemotherapy [2]. The German rectal study [3], which established pre-operative chemoradiation as a standard of care for stage II-III rectal adenocarcinoma, showed high rates of distant recurrence at 10 years (30%) compared to local recurrence (7%). It has been hypothesized that higher rates of distant recurrence may be the result of delays in treatment with systemic therapy and poor compliance in the adjuvant setting. Previous studies have demonstrated that more than half of patients receive no or incomplete courses of chemotherapy following surgery, primarily due to disease progression, patient refusal, and postoperative complications which occur in approximately 20% of patients [4,5].

Numerous studies have shown that pathologic tumor response rates following nCRT for rectal cancer are an important prognostic factor for local and distant disease-free survival [6,7]. This is particularly true for rates of pathologic nodal response (ypN) where posttreatment pathology stage (yp) N0, N1, and N2 are associated with 10-year disease-free survival rates of 84%, 59%, and 28%, respectively [8].

Total neoadjuvant therapy (TNT), consisting of induction chemotherapy followed by concurrent chemoradiation and surgical resection, is a therapeutic strategy proposed to better target micrometastatic disease through early exposure to high dose chemotherapy and increased compliance with systemic treatment [9]. Induction chemotherapy in this setting is typically an oxaliplatin-based, multi-agent regimen administered for six to eight cycles prior to concurrent chemoradiation as seen in currently ongoing clinical trials NRG GI-002 (clinicaltrials.gov, NCT 02921256) and PROSPECT (clinicaltrials.gov, NCT 01515787). The use of TNT has been shown to improve pathologic complete response (pCR) rates, however, there is a lack of data examining whether or not this directly translates into improved disease outcomes [9,10]. We utilized the National Cancer Database (NCDB) to evaluate the effect of TNT on pathologic treatment response and to determine whether or not this translates into a survival benefit. We also sought to determine the clinical factors associated with the utilization of TNT.

## Materials and methods

Patient selection

**Figure 1:**
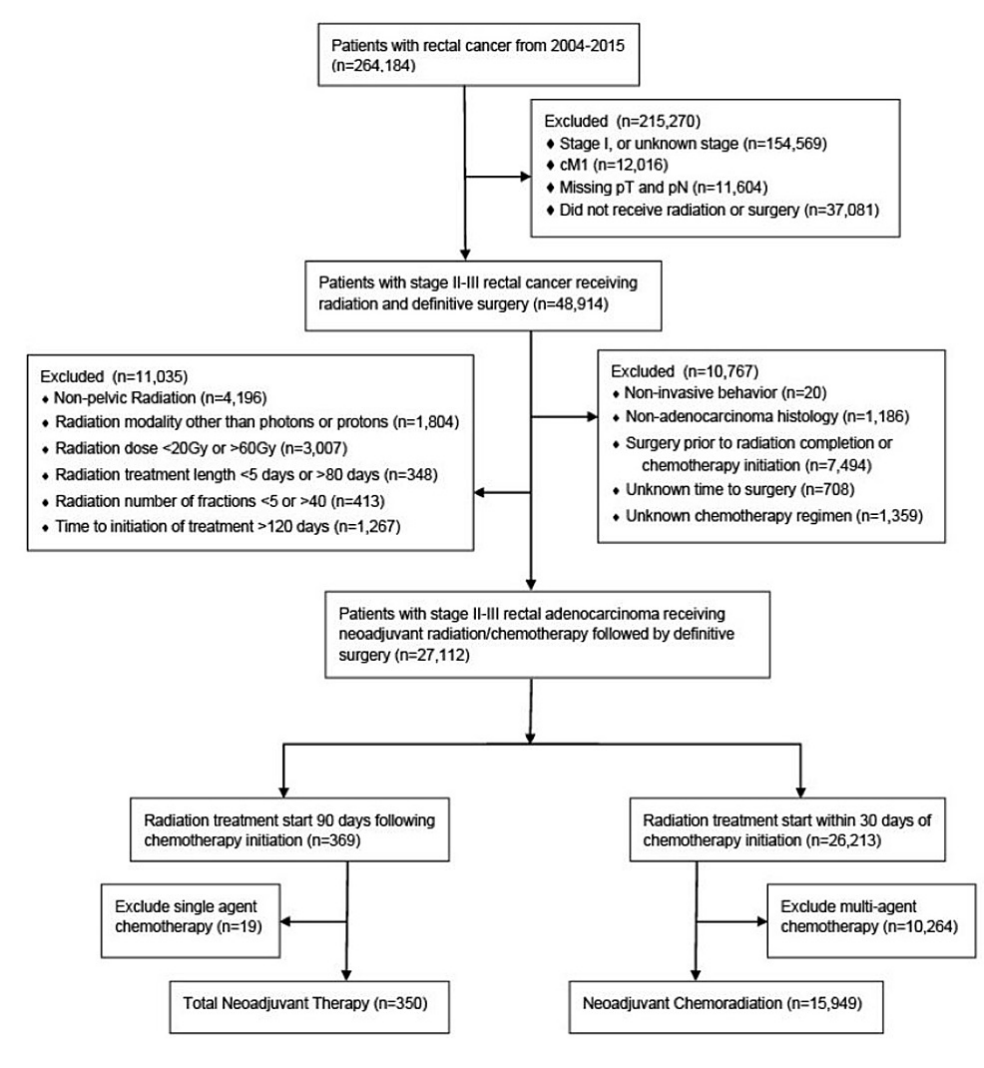
Consolidated Standards of Reporting Trials (CONSORT) diagram showing patient selection

We utilized the data set of the National Cancer Database (NCDB) to identify our study population consisting of patients diagnosed with rectal cancer from 2004-2015. The institutional review board deemed this study exempt due to the use of de-identified, population-based patient data. A consolidated standard of reporting trials (CONSORT) diagram is provided to show the selection criteria (Figure 1). Patients were excluded if they had stage I disease (based on the provided American Joint Committee on Cancer [AJCC] staging), metastatic disease, or incomplete clinical and pathologic staging information. We further excluded patients that did not undergo radiation treatment or definitive surgery. We then excluded patients with non-adenocarcinoma histology, if it was not known whether they received single or multi-agent chemotherapy, if surgery was performed prior to radiation or chemotherapy initiation, or if the length of time from diagnosis to surgery was unknown as we would be unable to determine the sequencing of treatment. We also excluded patients who were treated with non-standard radiation doses (<20Gy or >60Gy) or fractionation regimens (<5 or >40 fractions), had prolonged delay from diagnosis to treatment initiation (>120 days), were treated with a radiation modality other than protons or photons, or had limited post-surgical follow-up within one month of their surgery date to account for immortal time bias. This resulted in 27,112 patients prior to treatment stratification.

The remaining patients were then stratified into two treatment groups: the TNT group and the nCRT group. Patients starting radiation therapy >90 days after initiating multi-agent chemotherapy were included in the TNT group, whereas those starting radiation treatment within 30 days of single-agent chemotherapy were included in the nCRT group. After applying all exclusion criteria, 350 patients in the TNT group and 15,949 patients in the nCRT group were included in the final analysis. Pathologic response was determined using clinical and pathological staging information.

Statistics

The primary outcome was overall pCR rate, as well as the pCR rate of the primary tumor (ypT0) and nodes (ypN0). We also evaluated overall survival (OS) which was calculated from the date of diagnosis to the date of last contact or death, as we are unable to directly assess the distant metastasis rate using the NCDB dataset. Additional factors examined include patient age, gender, insurance coverage, residence income data, urban or rural residence, academic versus non-academic treatment facility, Charlson/Deyo comorbidity index, year of diagnosis, grade, clinical and pathologic T and N stage based on the AJCC eighth edition, and neoadjuvant rectal cancer (NAR) score. The NAR score is a composite score that predicts overall survival after neoadjuvant treatment for rectal cancer and is based on the pathologic response to therapy [11,12]. A NAR score >16 falls into the high-risk category and is associated with worse overall survival compared to scores <16. We also evaluated the time from radiation treatment completion to surgery, the total radiation dose administered, length of radiation treatment in days, and time from diagnosis to initiation of chemotherapy. We dichotomized groups based on the median value.

Baseline patient and treatment characteristics were compared between TNT and nCRT groups using χ2 test. Bivariate logistic regression models were used to evaluate the association between independent variables of interest. Variables that were significant on univariate analysis were included in a multivariate binomial regression analysis. These models were also used to evaluate predictors of pCR within each treatment group. The Kaplan-Meier method was used to assess survival outcomes, and the log-rank test was used to assess statistical significance between groups. The factors that were statistically significant on univariate analysis were entered using stepwise selection for multivariate analysis using Cox proportional hazard models to calculate adjusted hazard ratios for survival.

Propensity score analysis was conducted to account for the lack of randomization between the treatment groups. Variables found to be significantly associated with TNT use on univariate logistic regression were included. Overall survival was then determined using the Cox proportional hazards model adjusting for propensity score. The matched groups were balanced based on a standardized difference of <0.1 between factors. Any p-value less than 0.05 was considered statistically significant. The hazard ratios (HRs), odds ratios (ORs), and 95% confidence intervals (CIs) are reported. Statistical analysis was performed using IBM SPSS version 24 (IBM cooperation, Armonk, NY).

## Results

We identified a total of 16,299 patients diagnosed with locally advanced rectal adenocarcinoma meeting our inclusion criteria, with 15,949 in the nCRT group, and 350 in the TNT group (Figure 1). Baseline patient characteristics are outlined in Table 1. The median patient age was 54 years (Interquartile range (IQR): 46-63) in the TNT group and 62 years (IQR: 53-71) in the nCRT group. The distribution of gender, insurance coverage, and tumor grade were similar between the groups. Patients treated in the TNT group were more likely to be younger (p<0.001), treated at an academic facility (p<0.001), have a higher income (p<0.001), have a lower comorbidity score (p<0.001), and more recent treatment (p<0.001). There were proportionally more patients with cT4 disease (17% vs 8%), node-positive disease (81% vs 50%), and cN2 disease (20% vs 7%), in the TNT group versus the nCRT group, respectively.

**Table 1:** Baseline patient characteristics nCRT: neoadjuvant chemoradiation; TNT: total neoadjuvant therapy

Characteristic	nCRT (N=15,949) n (%)	TNT (N=350) n (%)	P-value
Age (years)			<0.001
≤ 65	9594 (60%)	288 (82%)	
> 65	6355 (40%)	62 (18%)	
Gender			0.62
Male	9914 (62%)	213 (61%)	
Female	6035 (38%)	137 (39%)	
Facility			<0.001
Community	8083 (51%)	55 (16%)	
Academic	7301 (46%)	256 (73%)	
Unknown	565 (3%)	39 (11%)	
Insurance			0.15
Uninsured	744 (5%)	9 (3%)	
Insured	15017 (94%)	338 (96%) 3 (1%)	
Unknown	188 (1%)		
Income ($)		79 (23%)	<0.001
< 48,000	6763 (42%)	271 (77%)	
≥ 48,000	9055 (57%)	0 (0%)	
Unknown	131 (1%)		
Population		308 (88%)	
Urban	15167 (95%)	5 (1%)	
Rural	419 (3%)	37 (11%)	<0.001
Unknown	363 (2%)		
Comorbidity Score		301 (86%)	
0	12358 (78%)	49 (14%)	
≥ 1	3591 (22%)		<0.001
Year		99 (28%)	
2004-2012	9461 (59%)	251 (72%)	
2013-2015	6488 (41%)		<0.001
Grade		277 (79%)	
1-2	11890 (75%)	36 (10%)	
3	1688 (10%)	37 (11%)	0.072
Unknown	2371 (15%)		
Clinical T stage		18 (5%)	
cT1-T2	800 (5%)	264 (76%)	
cT3	13684 (86%)	60 (17%)	<0.001
cT4	1249 (8%)	8 (2%)	
Unknown	216 (1%)		
Clinical N stage		64 (18%)	
cN0	7602 (48%)	215 (61%)	
cN1	6851 (43%)	69 (20%)	<0.001
cN2	1141 (7%)	2 (1%)	
Unknown	355 (2%)		

The treatment characteristics for patients are outlined in Table 2. The median time from radiation treatment completion to surgery was 61 days for the TNT group and 56 days for the nCRT group (OR=1.49, p<0.001), which was not significant on multivariate analysis (p=0.6). The median total radiation dose (50.4 Gy) and time to initiation of chemotherapy (33 days) were similar between groups. A majority of patients had a total radiation dose of between 50Gy to 54Gy with 72.6% vs. 77% in the TNT and nCRT groups, respectively. Short course radiation to 25Gy was administered for 2.6% of patients in the TNT group and 0.2% of patients in the nCRT group. Radiation doses >50.4 were used to treat 8% vs. 13% of patients in the TNT and nCRT groups, respectively (OR=0.58, p=0.005), although this was not significant on multivariate analysis (OR=0.69, p=0.08).

**Table 2:** Treatment characteristics nCRT: neoadjuvant chemoradiation; TNT: total neoadjuvant therapy; OR: odds ratio; CI: confidence interval; pCR: pathologic complete response; NAR: neoadjuvant rectal cancer

Treatment Characteristics						Propensity Match	
Characteristic		nCRT (N=15,949)	TNT (N=350) n (%)	OR [95% CI]	P-value	OR [95% CI]	P-value
		n (%)					
Pathologic T Stage							
ypT0		2372 (15%)	64 (18%)	reference			
ypT1		1200 (8%)	23 (7%)	0.71 [0.44-1.15]	0.16		
ypT2		4215 (26%)	98 (28%)	0.86 [0.63-1.19]	0.36		
ypT3		6991 (44%)	141 (40%)	0.75 [0.55-1.01]	0.06		
ypT4		677 (4%)	19 (5%)	1.04 [0.62-1.75]	0.88		
Unknown		494 (3%)	5 (2%)	0.38 [0.15-0.94]	0.04		
Pathologic T Response							
ypT+		12889 (85%)	275 (82%)	reference		reference	
ypT0		2357 (15%)	62 (18%)	1.23 [0.93-1.63]	0.14	1.26 [0.94-1.68]	0.13
Pathologic N Stage							
ypN0		11272 (71%)	258 (73%)	reference			
ypN1		3171 (20%)	59 (17%)	0.81 [0.61-1.08]	0.16		
ypN2		1183 (7%)	31 (9%)	1.15 [0.79-1.67]	0.48		
Unknown		323 (2%)	2 (1%)	0.27 [0.07-1.09]	0.07		
Pathologic N Response							
ypN+		2848 (36%)	79 (28%)	reference		reference	
ypN0		5024 (64%)	204 (72%)	1.46 [1.12-1.91]	0.005	1.53 [1.16-2.00]	0.003
pCR							
ypT+ or ypN+		12981 (85.8%)	283 (82.5%)	reference		reference	
ypT0N0		2151 (14.2%)	60 (17.5%)	1.28 [0.97-1.70]	0.087	1.34 [1.00-1.80]	0.053
Pathologic Response							
ypT+N+		2601 (35%)	71 (26%)	reference			
ypT0N+		96 (1%)	4 (1%)	1.53 [0.55-4.27]	0.42		
ypT+N0		3769 (51%)	148 (55%)	1.44 [1.08-1.92]	0.013		
ypT0N0		993 (13%)	48 (18%)	1.77 [1.22-2.57]	0.003		
NAR Score							
<8		3299 (21%)	90 (27%)	reference			
8-16		7483 (49%)	163 (48%)	0.80 [0.62-1.04]	0.09		
>16		4632 (30%)	87 (25%)	0.69 [0.51-0.93]	0.014		
Radiation Completion to Surgery (days)							
≤ 56		8089 (51%)	143 (41%)	reference			
> 56		7860 (49%)	207 (59%)	1.49 [1.20-1.85]	<0.001		
Total Dose (Gy)							
≤ 50.4		13802 (87%)	321 (92%)	reference			
> 50.4		2147 (13%)	29 (8%)	0.58 [0.40-0.85]	0.005		
Length Radiation Treatment (days)							
≤ 40		9320 (58%)	256 (73%)	reference			
> 40		6629 (42%)	94 (27%)	0.52 [0.41-0.66]	<0.001		
Diagnosis to Chemo Initiation (days)							
≤ 33		7983 (50%)	186 (53%)	reference			
> 33		7966 (50%)	164 (47%)	0.88 [0.72-1.09]	0.25		

There was a trend towards a better pCR rate in the TNT group (17.5% vs 14.2%, p=0.053 on propensity-matched analysis [PMA]). The rate of complete pathologic response of the primary tumor (ypT0) was similar in the TNT and nCRT groups (18% vs 15%, respectively, p=0.13), however, the rate of nodal pathologic complete response (ypN0) was significantly improved for those receiving TNT on both univariate and PMA (72% vs 64%, p=0.003) (Table 2). The rate of nodal pCR for all patients was 65.3% in those with N1 disease, and 57.3% for patients with N2 disease. When stratified by treatment group, the nCRT group had lower rates of nodal pCR for those with N2 disease versus N1 disease (OR=0.7, p<0.001), whereas nodal pCR rates did not differ between N1 and N2 patients in the TNT group (OR=0.78, p=0.40). Patients in the TNT group were less likely to have a high risk NAR score (>16) compared to the nCRT group (25% vs. 30%, respectively, p=0.014).

For the overall study population, improved overall pCR rates were associated with treatment at an academic facility, higher income, being insured, and more recent treatment. The total dose and length of radiation treatment had no effect on pCR, however, time from radiation completion to surgery of >56 days was associated with improved pCR (OR=1.21, p<0.001). High tumor grade (12.8% for grade 1-2 and 9.2% for grade 3, p<0.001), higher T stage (14.7% for stage T3 and 7.1% for T4), and increasing nodal burden (13.5% for N1 and 11.8% for N2) were associated with worse pCR.

Factors associated with pCR stratified by treatment group are shown in Table 3. For patients receiving nCRT, pretreatment stage T3/T4 disease, increasing nodal burden (N1, OR=0.83; N2, OR=0.69), and longer time from diagnosis to treatment initiation and radiation completion to surgery were associated with worse overall pCR rates. For patients receiving TNT, only cT4 disease was associated with worse overall pCR rates (OR=0.2, p=0.02).

**Table 3:** Patient and treatment characteristics associated with pCR stratified by treatment group nCRT: neoadjuvant chemoradiation; TNT: total neoadjuvant therapy; OR: odds ratio; CI: confidence interval; pCR: pathologic complete response; NAR: neoadjuvant rectal cancer

Characteristic	nCRT group		TNT group	
	OR [95% CI]	P-value	OR [95% CI]	P-value
Age (years)				
≤ 65	reference		reference	
> 65	1.06 [0.96-1.16]	0.24	1.43 [0.71-2.84]	0.32
Gender				
Male	reference		reference	
Female	1.07 [0.98-1.18]	0.13	1.64 [0.93-2.87]	0.09
Facility				
Community	reference		reference	
Academic	1.12 [1.02-1.23]	0.02	1.10 [0.50-2.41]	0.82
Insurance				
Uninsured	reference		reference	
Insured	1.53 [1.19-1.96]	0.001	1.51 [0.18-12.53]	0.7
Income ($)				
< 48,000	reference		reference	
≥ 48,000	1.18 [1.08-1.30]	<0.001	1.33 [0.65-2.70]	0.43
Population				
Urban	reference		reference	
Rural	0.64 [0.46-0.89]	0.009	1.32 [0.15-12.09]	0.8
Comorbidity Score				
0	reference		reference	
≥ 1	0.95 [0.85-1.06]	0.32	1.14 [0.52-2.50]	0.75
Year				
2004-2012	reference		reference	
2013-2015	1.39 [1.27-1.53]	<0.001	0.98 [0.53-1.82]	0.95
Grade				
1-2	reference		reference	
3	0.67 [0.56-0.80]	<0.001	1.77 [0.78-4.02]	0.17
Clinical T stage				
T1-T2	reference		reference	
T3	0.65 [0.54-0.78]	<0.001	0.40 [0.14-1.13]	0.08
T4	0.28 [0.21-0.37]	<0.001	0.20 [0.06-0.75]	0.02
Clinical N stage				
N0	reference		reference	
N1	0.83 [0.75-0.91]	<0.001	1.13 [0.53-2.43]	0.75
N2	0.69 [0.56-0.84]	<0.001	1.23 [0.50-3.04]	0.65
Radiation Completion to Surgery (days)				
≤ 56	reference		reference	
> 56	1.22 [1.12-1.34]	<0.001	0.80 [0.46-1.40]	0.44
Total Dose (Gy)				
≤ 50.4	reference		reference	
> 50.4	0.94 [0.82-1.07]	0.34	0.52 [0.15-1.78]	0.3
Length Radiation Treatment (days)				
≤ 40	reference		reference	
> 40	0.97 [0.88-1.06]	0.47	0.99 [0.53-1.86]	0.98
Diagnosis to Chemo Initiation (days)				
≤ 33	reference		reference	
> 33	1.29 [1.17-1.41]	<0.001	1.26 [0.72-2.20]	0.42

**Figure 2:**
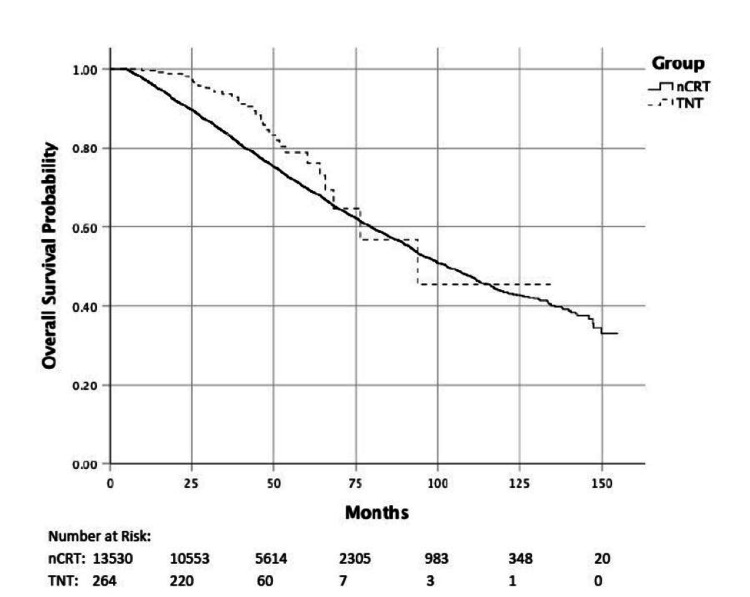
Overall survival by treatment group

Median follow-up was 38 months (TNT group) versus 54 months (nCRT group). The five-year OS was significantly better for the TNT group (76.2% vs. 69.9%) on univariate analysis (HR=0.58, p=0.002), but not on PMA (adjusted HR=0.79, p=0.19) (Table 4, Figure 2).

**Table 4:** Univariate Cox proportional hazards model for overall survival nCRT: neoadjuvant chemoradiation; TNT: total neoadjuvant therapy; HR: hazard ratio; CI: confidence interval; pCR: pathologic complete response; NAR: neoadjuvant rectal cancer

Prognostic Factor	HR [95% CI]		P-value	Propensity Matched HR [95% CI]	P-Value
Group					
nCRT	reference			reference	
TNT	0.58 [0.41-0.82]	0.002		0.79 [0.56-1.12]	0.19
Age (years)					
≤ 65	reference				
> 65	1.84 [1.73-1.96]	<0.001			
Gender					
Male	reference				
Female	0.84 [0.79-0.90]	<0.001			
Facility					
Community	reference				
Academic	0.81 [0.76-0.86]	<0.001			
Insurance					
Uninsured	reference				
Insured	0.91 [0.78-1.05]	0.19			
Income ($)					
< 48,000	reference				
≥ 48,000	0.84 [0.78-0.89]	<0.001			
Population					
Urban	reference				
Rural	1.04 [0.85-1.26]	0.72			
Comorbidity Score					
0	reference				
≥ 1	1.47 [1.37-1.58]	<0.001			
Year					
2004-2012	reference				
2013-2015	0.84 [0.76-0.93]	0.001			
Grade					
1-2	reference				
3	1.61 [1.48-1.76]	<0.001			
Clinical T stage					
T1-T2	reference				
T3	1.29 [1.09-1.52]	0.002			
T4	2.19 [1.81-2.64]	<0.001			
Clinical N stage					
N0	reference				
N1	0.98 [0.91-1.04]	0.49			
N2	1.23 [1.07-1.40]	0.003			
Pathologic T Stage					
pT0	reference				
pT1	1.23 [1.02-1.47]	0.026			
pT2	1.35 [1.19-1.55]	<0.001			
pT3	2.48 [2.20-2.80]	<0.001			
pT4	4.63 [3.95-5.43]	<0.001			
Pathologic T Response					
pT+	reference				
pT0	0.48 [0.43-0.54]	<0.001			
Pathologic N Stage					
pN0	reference				
pN1	1.70 [1.58-1.84]	<0.001			
pN2	2.73 [2.48-2.99]	<0.001			
Pathologic N Response					
pN+	reference				
pN0	0.47 [0.43-0.52]	<0.001			
pCR					
ypT+ or ypN+	reference				
ypT0N0	0.46 [0.40-0.52]	<0.001			
Pathologic Response					
ypT+N+	reference				
pT0N+	0.39 [0.24-0.66]	<0.001			
pT+N0	0.49 [0.45-0.54]	<0.001			
pT0N0	0.31 [0.25-0.38]	<0.001			
NAR Score					
<8	reference				
8-16	1.53 [1.38-1.70]	<0.001			
>16	2.60 [2.34-2.89]	<0.001			
Radiation Completion to Surgery (days)					
≤ 56	reference				
> 56	1.16 [1.09-1.24]	<0.001			
Total Dose (Gy)					
≤ 50.4	reference				
> 50.4	1.11 [1.02-1.21]	0.018			
Length Radiation Treatment (days)					
≤ 40	reference				
> 40	1.23 [1.15-1.31]	<0.001			
Diagnosis to Chemo Initiation (days)					
≤ 33	reference				
> 33	1.01 [0.95-1.08]	0.76			

**Figure 3:**
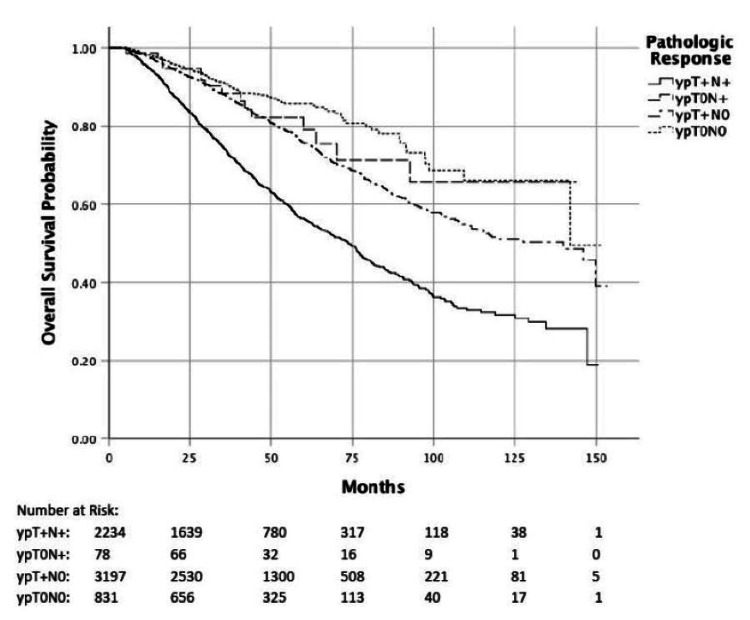
Predicted survival based on pathologic response to therapy

Older age, a co-morbidity score ≥1, high-grade disease, and advanced T and N stage were associated with worse overall survival (p<0.001), whereas female gender, treatment at an academic facility, and higher income were associated with improved survival (Table 4). The five-year OS for patients with a pathologic complete response versus residual disease was 85.1% vs. 67.5%, respectively (HR=0.46, p<0.001). Pathologic complete nodal (ypT+N0) and primary tumor response (ypT0N+) were both associated with improved survival compared to residual primary tumor and nodal disease (ypT+N+) (Table 4). There was no difference in survival between pCR of the primary tumor versus nodal disease (HR=1.26, 95%CI: 0.75-2.10, p=0.38). Figure 3 demonstrates overall survival based on pathologic response to treatment.

When stratified by clinical stage, we found that patients with cT3 or cN1 disease (adjusted HR=0.6, p=0.038, and adjusted HR=0.58, p=0.049, on PMA, respectively) had improved OS when treated with TNT. Patients with cT4 disease or cN2 disease were not found to have improved OS with TNT (p=0.853 vs. p=0.791, respectively) (Table 5). 

**Table 5:** Overall survival stratified by clinical stage nCRT: neoadjuvant chemoradiation; TNT: total neoadjuvant therapy; HR: hazard ratio; CI: confidence interval

Group	cT3		cT4		cN1		cN2	
	Propensity Matched HR [95% CI]	P-value	Propensity Matched HR [95% CI]	P-value	Propensity Matched HR [95% CI]	P-value	Propensity Matched HR [95% CI]	P-value
nCRT	reference		reference		reference		reference	
TNT	0.60 [0.37-0.97]	0.038	0.94 [0.51-1.74]	0.853	0.58 [0.34-1.00]	0.049	0.91 [0.46-1.82]	0.791

## Discussion

We used the NCDB to evaluate a large patient population with locally advanced rectal cancer to investigate pCR rates and OS outcomes for patients treated with TNT or nCRT. As demonstrated in previous studies, we found that TNT was more commonly utilized for patients with advanced-stage cT4 disease (17% vs 8%) and node-positive disease (20% vs 7% for N2 disease). When evaluating treatment response, we found a trend towards improved pCR with the use of TNT (17.5% vs 14.2%, p =0.053), though this was not statistically significant. No statistically significant difference was observed in five-year OS between treatment groups (TNT 76.2% vs. nCRT 69.9%), despite higher utilization of TNT for more advanced-stage disease. This is consistent with other NCDB analyses demonstrating no difference in overall survival between treatment groups [13,14]. However, when stratified by clinical stage, we found that patients with cT3 or cN1 disease had better OS when treated with TNT versus nCRT. Patients with cT4 disease or cN2 disease were not found to have improved OS with TNT (p=0.853 vs. p=0.791, respectively).

When evaluating tumor characteristics associated with pathologic tumor response, there was a lower pCR rate in patients with cT4 disease in both treatment groups. In contrast, we found that increasing nodal burden was associated with a lower pCR rate for the nCRT group whereas it was independent of pCR rate in the TNT group. When evaluating nodal pCR, we found that the rate of pathologic complete nodal response was significantly improved with the use of TNT compared to nCRT (72% vs 64%). Several studies have demonstrated that pathologic tumor response rates are important prognostic factors for survival in rectal cancer [6-8,15-16], and are achieved in roughly 14-40% of patients treated with neoadjuvant therapy [17,18]. An exploratory analysis of the CAO/ARO/AIO-94 trial showed that both pathologic nodal response and primary tumor response after nCRT were the most important independent prognostic factors for disease-free, metastases-free, and local relapse-free survival [8]. In concordance with these results, we found that overall survival was better for patients who achieved a pathologic complete response (five-year OS of 85% vs. 67%).

The novel finding in our study was that TNT improves the rate of nodal pCR compared to nCRT and could account for the improved overall survival observed with TNT in the subset of patients with cT3 and cN1 disease. These findings suggest that preoperative TNT for locally advanced rectal cancer may provide the most benefit for patients with less advanced-stage disease. While there are existing clinical trials comparing these treatment modalities, long-term follow-up is needed to confirm a survival benefit in this subset of patients.

There are several inherent limitations associated with analyzing retrospective data from a large cancer registry, including uncertainties in the accuracy of collected data and the potential for selection bias. Further, data collected from the NCDB database does not include pertinent prognostic information, including tumor location and tumor distance from the mesorectal fascia, which recent studies have suggested may obviate the need for neoadjuvant therapy altogether for select patients [19].

## Conclusions

In our study, TNT was associated with higher nodal pCR rates, which is independently associated with improved overall survival. When compared to nCRT, TNT was associated with improved overall survival for patients with cT3 and cN1 disease. Our results suggest that while TNT is the treatment of choice for patients with more advanced disease, consideration should be made in utilizing this modality for patients with less advanced stage III rectal cancer.
